# Incidental detection of a retained left atrial catheter via intraoperative transesophageal echocardiography in a patient undergoing tricuspid valve replacement: A case report

**DOI:** 10.1097/MD.0000000000020058

**Published:** 2020-05-08

**Authors:** Taehee Pyeon, Hong-Beom Bae, Jeong Il Choi, Taeyeong Kim, Joungmin Kim

**Affiliations:** a Department of Anesthesiology and Pain Medicine, Chonnam University Hospital; b Department of Anesthesiology and Pain Medicine, Chonnam National University Medical School, Gwangju, South Korea.

**Keywords:** cardiac foreign body, indwelling catheter, left atrial catheter, transesophageal echocardiography

## Abstract

**Rationale:**

A cardiac foreign body can cause thrombosis or infection, but sometimes it may not cause any symptoms in a patient. The diagnosis is mainly performed using a radiological examination. Especially, ultrasound is useful not only for detecting the foreign body but also for hemodynamic findings. However, the disadvantage of ultrasound is that it cannot be used where shadows are generated because of poor permeability. The transesophageal echocardiography (TEE) is superior to transthoracic echocardiography (TTE) for identifying posterior cardiac structures because the probe is located in the esophagus behind the heart. Here, we report on the incidental finding of a foreign body in the left atrium through TEE during cardiac surgery. It did not cause any symptoms or signs for 20 years.

**Patient concerns:**

A 75-year-old female patient with severe tricuspid regurgitation underwent tricuspid valve replacement (TVR) under general anesthesia. She had a history of mitral valve replacement (MVR) and tricuspid annuloplasty surgery 20 years ago.

**Diagnosis:**

A hyper-echoic floating intracardiac foreign body was observed in the left atrium during TEE examination. It was not detected in the preoperative imaging studies such as X-ray, computed tomography, TTE.

**Interventions:**

The cardiac foreign body found using TEE was visually confirmed through an incision in the left atrium. A long and thin foreign body was located in the right upper pulmonary vein to the left atrium, which was considered to be a left atrial catheter used during the MVR surgery performed 20 years ago. After removing the foreign body, the planned TVR operation proceeded.

**Outcomes:**

After removing the intracardiac foreign body and TVR, the patient was admitted into the intensive care unit followed by the general ward as planned, and discharged without any complications.

**Lessons:**

TEE was very useful for diagnosing a foreign body in the posterior part of the heart. TEE performed during the perioperative period should be performed beyond the level of re-confirming the findings of TEE performed prior to surgery. If a retained catheter is detected, it may be appropriate to remove it considering the risk of complications.

## 1 Introduction

A case report of a cardiac foreign body is uncommon, and most such bodies enter the heart during trauma or a therapeutic procedure.^[1,2]^ A cardiac foreign body may not cause any symptoms in the patient for a long time, but it can cause complications such as repeated thrombosis or infection.^[1–4]^ A cardiac foreign body can be diagnosed using imaging methods such as X-ray, computed tomography (CT), or echocardiography.^[1,2]^ Among them, intraoperative transesophageal echocardiography (TEE) is considered to be the most effective diagnostic tool.^[5]^ The removal procedure for a cardiac foreign body should be determined by comparing the adverse effects of leaving it in with the side effects of removal. If the foreign body is a catheter, surgical removal may be considered rather than percutaneous removal to prevent fracturing the catheter.^[3]^ We report a case of a retained left atrial catheter that remained without symptoms after mitral valve replacement (MVR) surgery performed 20 years ago. The foreign body was diagnosed via intraoperative TEE during tricuspid valve replacement (TVR).

### 1.1 Ethical review

This case report was approved by the Clinical Ethics Committee of Chonnam National University Hospital, Gwangju, South Korea (CNUH-EXP-2019-074). The patient provided informed consent for the publication of the case.

## 2 Case presentation

**Figure 1:**
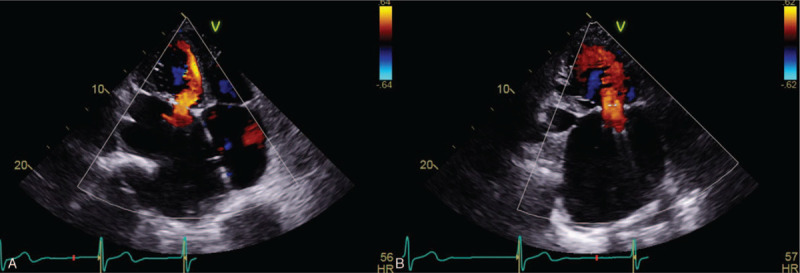
No specific findings were detected in the left atrium other than mitral regurgitation using preoperative transthoracic echocardiography. (A) Apical 4-chamber view. (B) Apical 2-chamber view.

**Figure 2:**
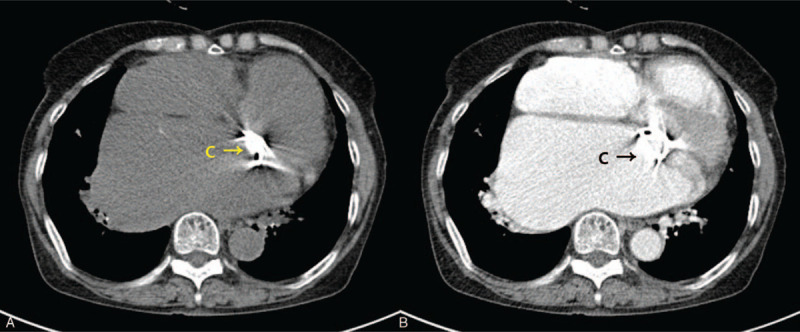
Preoperative CT scans revealed an enlarged atrium and a mechanical valve, but no specific findings in the left atrium. (A) Pre-enhanced CT scan at the pulmonary vein level. (B) Enhanced CT scan at the pulmonary vein level. (C) Previously inserted mitral prosthetic valve. CT = computed tomography.

A 75-year-old female visited the hospital with progressive severe dyspnea class III (New York Heart Association) for 1 year. She had a history of MVR and tricuspid annuloplasty (TAP) for mitral and tricuspid valve regurgitation 20 years ago. She had been taking warfarin, since the mechanical mitral valve was inserted. A chest X-ray showed marked cardiomegaly and evidence of a median sternotomy. Transthoracic echocardiography (TTE) revealed huge enlarged bi-atria without visible intracardiac thrombi, a mechanical mitral valve with mild paravalvular leakage, and severe tricuspid regurgitation (TR) due to coaptation failure with moderate pulmonary hypertension (Fig. 1). Coronary angiography showed no significant stenosis of the coronary arteries. A chest CT also revealed marked cardiomegaly and bi-atrial enlargement, evidence of a median sternotomy, and a replaced mechanical mitral valve (Fig. 2). No pulmonary abnormalities that cause dyspnea were detected on the chest CT. No abnormal laboratory findings were observed, including inflammatory markers. TVR was planned for severe TR under general anesthesia with cardiopulmonary bypass as an elective operation with heparin bridging therapy.

**Figure 3:**
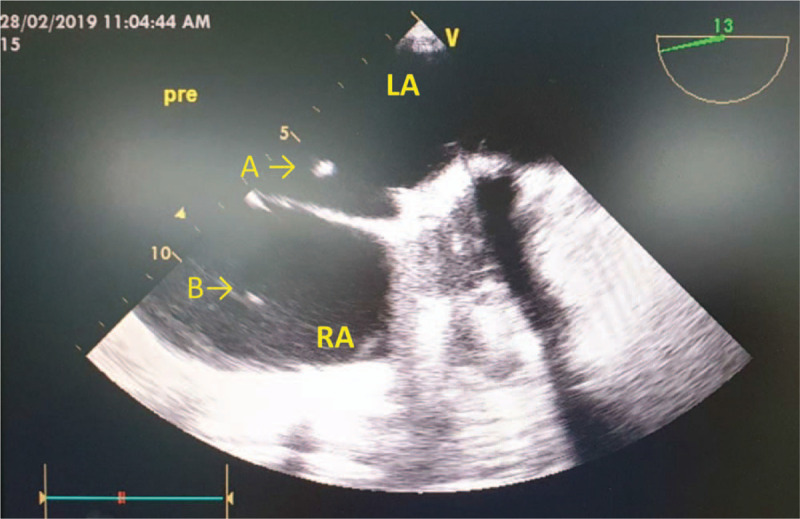
(A) A left atrial foreign body and (B) a Swan-Ganz catheter were observed in the right atrium using transesophageal echocardiography. LA = left atrium; RA = right atrium.

General anesthesia was induced uneventfully. The arterial line was inserted before induction of general anesthesia, and a Swan-Ganz catheter was inserted through the right internal jugular vein. We inserted the TEE probe to evaluate cardiac status preoperatively. We identified almost the same findings as with the previous TTE. However, a floating, 1-mm-diameter hyperechoic signal was found in the left atrium, which was not mentioned on the preoperative TTE (Fig. 3). We discussed the existence of an intracardiac foreign body with the cardiac surgeon, and the surgeon performed an additional left atrial incision to identify the foreign material prior to the main operation.

**Figure 4:**
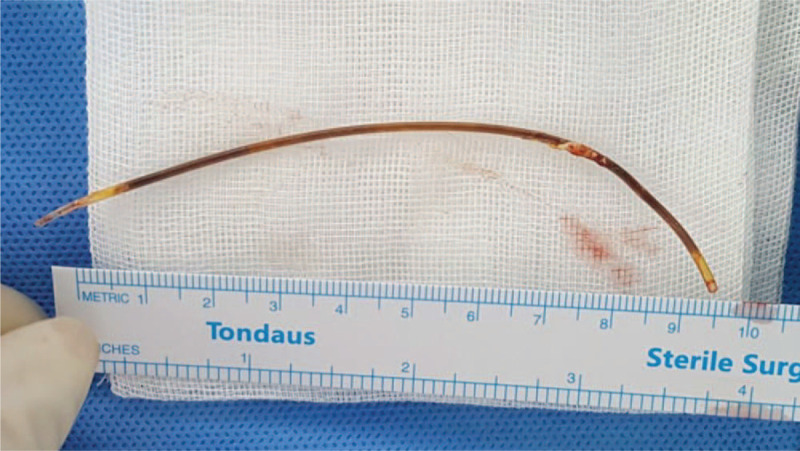
Foreign body in the heart removed by surgery. This is thought to be a left atrial catheter inserted 20 years ago. It had changed color, but no thrombosis was noted.

A long thin foreign body was observed in the right pulmonary vein to the left atrium. It was tied at the right upper pulmonary vein. The diameter of the foreign body was 1 mm, and its length was 13 cm (Fig. 4). It was presumed to be a catheter for measuring left atrial pressure at the time of the MVR surgery 20 years ago. The catheter had changed color, but the surface was smooth without blood clots. After removing the intracardiac foreign body, elective TVR was performed without any adverse events. After the operation, the patient was transferred to the intensive care unit as planned. She was transferred to the general ward on postoperative day 4 and was discharged on postoperative day 16.

## 3 Discussion

In our case, the intracardiac foreign body was a left atrial catheter. Left atrial catheters are used to measure left atrial pressure, take a blood sample, and inject drugs.^[6]^ These catheters are inserted through the right upper pulmonary vein and are occasionally tied to prevent dislocation.^[7]^ The left atrial catheter is usually removed at the end of surgery, but sometimes it is temporarily maintained for hemodynamic monitoring of the patient after a cardiac operation. In this case, part of the catheter was likely cut off during removal. Retained left atrial catheters have been reported to result in serious complications.^[3,8]^ For example, Yeo et al reported a cerebral vascular accident due to a retained left atrial catheter,^[3]^ whereas Erummond-Webb et al reported occlusion of a branch of the retinal artery due to a left atrial catheter 21 years after the cardiac operation.^[9]^

The most common symptom of a cardiac foreign body is dyspnea. However, it can cause other complications such as infection, pericardial effusion, cardiac tamponade, thrombosis, and arrhythmia. Intracardiac foreign bodies are symptomatic in 56% of patients and larger devices result in more severe complications.^[2]^ In some cases, an intracardiac foreign body may not cause any symptoms.

The patient in this report was asymptomatic for 20 years despite the relatively large foreign body located in the left atrium. The use of warfarin after inserting the mechanical valve is considered to be the cause of the patient's asymptomatic condition without a thrombotic event until endothelialization of the foreign body. As the catheter was tied to the pulmonary vein, it probably would have caused limited mechanical damage, such as cardiac perforation. No infection was evident, as the catheter was inserted under sterile conditions and antibiotic treatment was applied after the MVR surgery.

A foreign body in the heart is not common, but it has been reported to enter through various pathways. A catheter can be inserted directly into the heart or transferred to the heart through the systemic circulation. Trauma can induce an intracardiac foreign body when the heart is directly injured by a needle or a piece of metal.^[1]^ Therapeutic materials that are inserted directly into the heart, such as pacemaker leads^[10]^ or a Swan-Ganz catheter,^[11]^ can be left in the heart by mistake. A foreign body inserted at other parts of the body via trauma can be transferred into the heart by the systemic circulation.^[12]^ In addition, therapeutic materials, such as inferior vena cava filters,^[13]^ cement for vertebroplasty,^[14]^ and Kirshner wires used for fractures,^[15]^ can travel to the heart through the systemic circulation.

In our case, the cardiologist performed TTE several times after MVR surgery, but he did not find a foreign body. TTE is more useful than TEE to inspect anterior cardiac structures, such as the right ventricle, right ventricular outflow tract, pulmonary valve, and anterior pericardium, but not posterior cardiac structures, such as the left atrium, mitral valve, subvalvular apparatus, interatrial septum, and left atrial appendage.^[16]^ A left atrial catheter or other radiolucent foreign body may not be found on a plain chest radiograph or a CT scan. Smith et al reported a missing left atrial catheter tip that was not seen using TTE or a plain chest radiograph, but was confirmed using TEE.^[5]^

## 4 Conclusions

A cardiac foreign body may be asymptomatic for a long time but can cause serious complications. Therefore, removing an intracardiac foreign body should be considered and followed up. TEE is a good modality to examine the posterior structure of the heart. If a foreign body in the heart is suspected but not visible through another test, TEE may be an excellent choice. Intraoperative TEE played a role as a new diagnostic test by re-confirming the preoperative findings.

## Author contributions

**Data curation:** Taeyeong Kim.

**Investigation:** Taehee Pyeon.

**Project administration:** Joungmin Kim.

**Supervision:** Hong-Beom Bae.

**Validation:** Jeong Il Choi.

**Writing – original draft:** Taehee Pyeon.

**Writing – review & editing:** Joungmin Kim.
